# Exploring the role of mitochondrial uncoupling protein 4 in brain metabolism: implications for Alzheimer’s disease

**DOI:** 10.3389/fnins.2024.1483708

**Published:** 2024-09-24

**Authors:** Simone M. Crivelli, Aisylu Gaifullina, Jean-Yves Chatton

**Affiliations:** Department of Fundamental Neurosciences, University of Lausanne, Lausanne, Switzerland

**Keywords:** mitochondria, uncoupling agent, uncoupling protein, UCP4, astrocytes, neurons, Alzheimer’s disease

## Abstract

The brain’s high demand for energy necessitates tightly regulated metabolic pathways to sustain physiological activity. Glucose, the primary energy substrate, undergoes complex metabolic transformations, with mitochondria playing a central role in ATP production via oxidative phosphorylation. Dysregulation of this metabolic interplay is implicated in Alzheimer’s disease (AD), where compromised glucose metabolism, oxidative stress, and mitochondrial dysfunction contribute to disease progression. This review explores the intricate bioenergetic crosstalk between astrocytes and neurons, highlighting the function of mitochondrial uncoupling proteins (UCPs), particularly UCP4, as important regulators of brain metabolism and neuronal function. Predominantly expressed in the brain, UCP4 reduces the membrane potential in the inner mitochondrial membrane, thereby potentially decreasing the generation of reactive oxygen species. Furthermore, UCP4 mitigates mitochondrial calcium overload and sustains cellular ATP levels through a metabolic shift from mitochondrial respiration to glycolysis. Interestingly, the levels of the neuronal UCPs, UCP2, 4 and 5 are significantly reduced in AD brain tissue and a specific UCP4 variant has been associated to an increased risk of developing AD. Few studies modulating the expression of UCP4 in astrocytes or neurons have highlighted protective effects against neurodegeneration and aging, suggesting that pharmacological strategies aimed at activating UCPs, such as protonophoric uncouplers, hold promise for therapeutic interventions in AD and other neurodegenerative diseases. Despite significant advances, our understanding of UCPs in brain metabolism remains in its early stages, emphasizing the need for further research to unravel their biological functions in the brain and their therapeutic potential.

## Graphical abstract

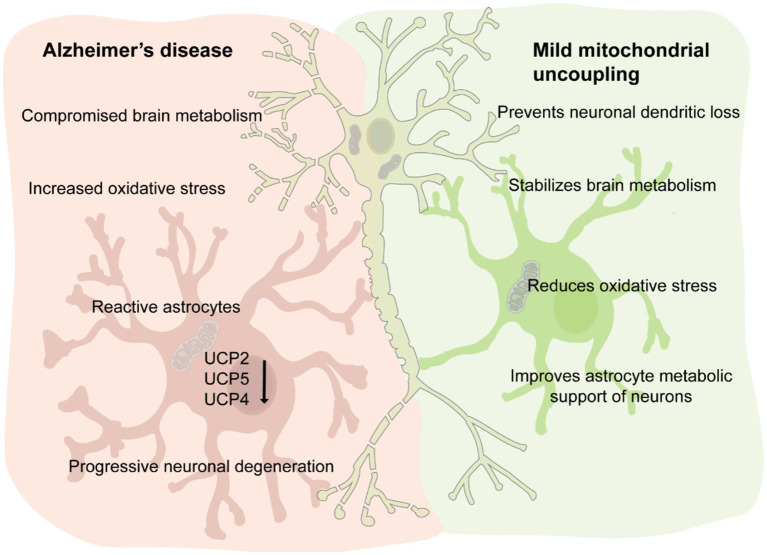

## 1 Background

### 1.1 Energy dynamics in the brain

Physiological brain activity requires sustained and highly regulated local flow of energy which is mostly derived from glucose metabolism (Kapogiannis and Avgerinos, 2020). Glucose enters the brain parenchyma from the blood vessels via specific transporters (GLUTs) located in the cell membrane of endothelial cells and astrocytes endfoot. Once taken-up by brain cells glucose is catabolized to produce energy via a coordinated network of metabolic pathways that include: i) the glycolytic pathway (glycolysis), ii) the tricarboxylic acid cycle (TCA; also called the Krebs cycle or citric acid cycle), and iii) oxidative phosphorylation. Glucose is metabolized by glycolysis to pyruvate, which enters mitochondria to undergo further transformation in the TCA cycle. Alternatively, glucose once converted to glucose 6-phosphate can enter the pentose phosphate pathway which controls oxidative stress via the production of 1,4-dihydronicotinamide adenine dinucleotide (NADH) in a cell type-specific manner (Dringen et al., 2007). Mitochondria are double-membraned organelles that provide a wide variety of biochemical services to the cell. In the past few decades, the research on mitochondria has exploded in the area of human pathology propelled by the discovery of novel functions and advancement in methodologies for studying their function (Jacobs, 2023). These organelles provide most of the cellular adenosine triphosphate (ATP) demand by oxidative phosphorylation. Oxidative phosphorylation transfers electrons from macronutrients (like glucose) to oxygen harnessing high energy phosphate bonds in the form of ATP. Oxidative phosphorylation has two parts: the electron transport chain (ETC) and chemiosmosis (moving of protons across a biological membrane). The ETC is a collection of proteins bound to the inner mitochondrial membrane and cofactors, through which electrons travel via a series of redox reactions. During this process, protons are pumped from the mitochondrial matrix to the intermembrane space, building-up an electrochemical gradient defined as protonmotive force. Most of this potential (quantified in the range 150–200 mV) drives ATP synthesis, but a fraction of it is released as heat by proton uniport carriers, which tends to depolarize mitochondria. These proton uniport carriers are collectively termed mitochondrial uncoupling proteins (UCPs). UCPs are members of the family of mitochondrial carrier proteins (solute carrier family 25, SCL25), which is the largest solute transporter family in humans. SLC25 proteins provide transport for amino acids, inorganic ions, carboxyl acids, cofactors, fatty acid, and nucleotides across the mitochondrial inner membrane (Ruprecht and Kunji, 2020). Of notice, there are still about one third of the SCL25 transporter proteins with unknown substrate. In this review, we will focus on the biological role and properties of the UCP4 (or SLC25A27).

### 1.2 Bioenergetic crosstalk between astrocytes and neurons

**Figure 1:**
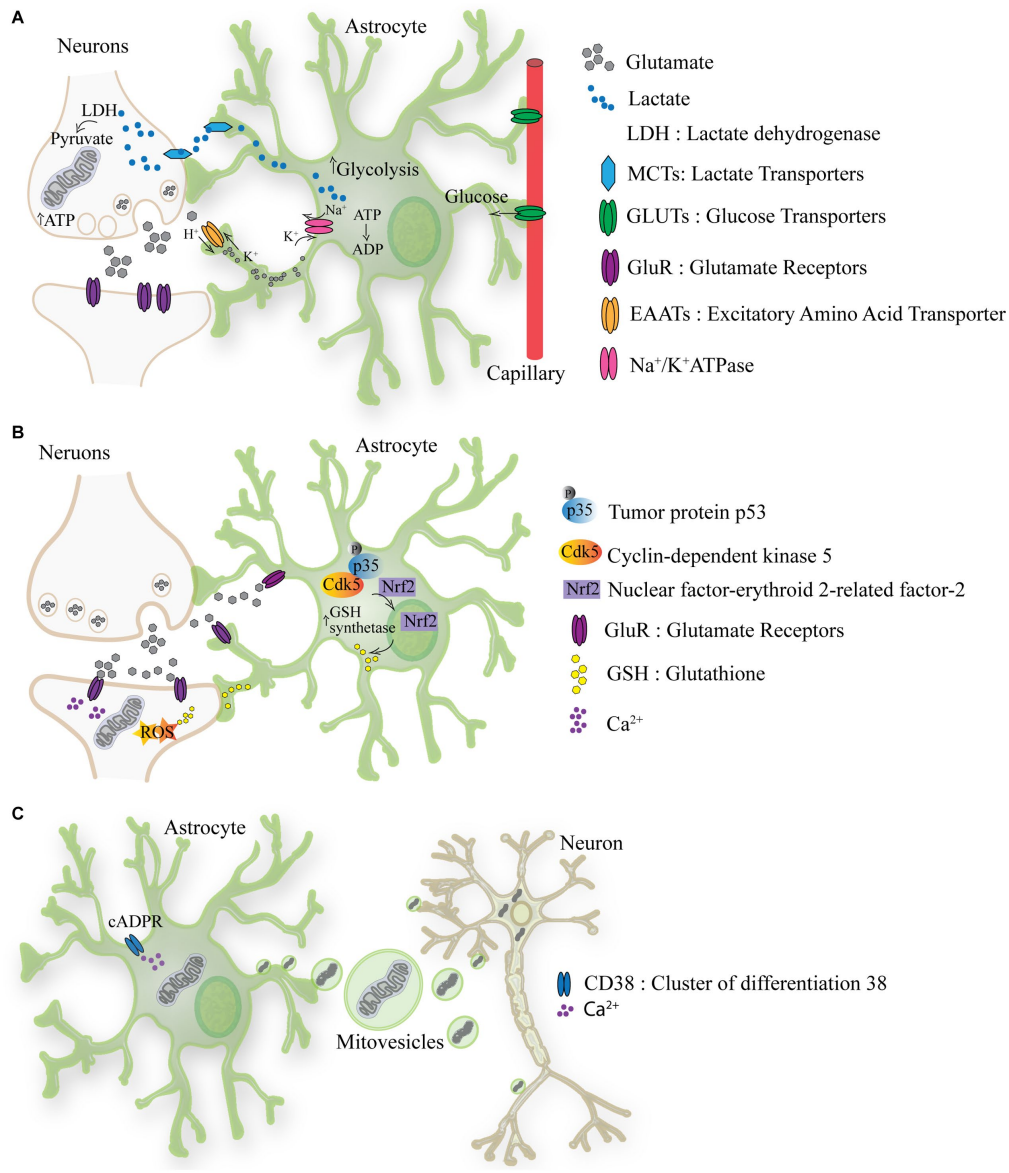
The metabolic crosstalk between astrocytes and neurons. **(A)** Lactate is produced from pyruvate by lactate dehydrogenase (LDH) an enzyme that operates in both directions, using NADH. Lactate produced by astrocytes can be used as energy currency in neurons, by entering the TCA cycle or as a substrate for the synthesis of glutamate. During intense neuronal activity extracellular glutamate rises leading to increased glutamate uptake by astrocytes through EAATs. This activates the Na+/K+ ATPase causing a consequential drop of ATP levels, which is counteracted by increasing glucose uptake from the blood stream and glycolysis. From glycolysis lactate is formed and released via MCTs to sustain and aid neurons. **(B)** Astrocyte-mediated redox adaptation to neurotransmission involves glutamate (Glu) released into the synaptic cleft, stimulating Glutamate receptors (Glu-R) in both post-synaptic neurons and astrocytes. Mitochondrial uptake of intracellular Ca^2+^ in post-synaptic neurons leads to reactive oxygen species (ROS) production. Glutamate binding to astrocytic receptors triggers a cascade via Cdk5-mediated phosphorylation of Nrf2, promoting its nuclear translocation and binding to antioxidant responsive elements (ARE). This prompts the expression of antioxidant genes, facilitating the biosynthesis and release of glutathione (GSH). Neurons uptake GSH precursors from astrocytes to detoxify activity-induced mitochondrial ROS, ensuring neuronal redox balance during synaptic activity. **(C)** The activation of CD38 (cluster of differentiation 38) by cyclic ADP-ribose (cADPR) induces the release of mitovesicles (extracellular vesicle containing mitochondria) by astrocytes which functionally support neurons. In turn mitochondria which require recycling are released by neurons and taken-up by astrocytes/glia.

Neurons convert glucose to pyruvate for the TCA cycle, meanwhile astrocytes play a more prominent role in glycolysis and lactate production, thereby supporting neuronal metabolism (Herrero-Mendez et al., 2009; Garcia-Nogales et al., 2003). Glycolysis, assessed as the rate of radioactive water formation from [3-^3^H] glucose is approximately four- to five times slower in neurons than in astrocytes (Herrero-Mendez et al., 2009). Hence, it appears that the neuronal capacity to perform glycolysis is limited, implying that neurons relies mostly on oxidative metabolism for energy production, via mitochondrial oxidative phosphorylation (Belanger et al., 2011). On the contrary, in astrocytes, glycolysis flux is strongly up-regulated. Enzymes like 6-phosphofructo-2-kinase which are key regulators of glycolysis are four times more active in astrocytes than in neurons (Almeida et al., 2004). Astrocytes store glycogen, which can be rapidly converted to pyruvate or lactate. Lactate is produced from pyruvate by lactate dehydrogenase (LDH) an enzyme that operates in both directions, using NADH as cofactor (Vaccari-Cardoso et al., 2022). Lactate can be used as an energy currency between astrocytes and neurons, by entering the TCA cycle or as a substrate for the synthesis of glutamate (Magistretti and Allaman, 2018). Neuronal activity depends on and regulates glycolysis in astrocytes. During intense neuronal activity extracellular glutamate rises leading to increased glutamate uptake by astrocytes. This activates the Na^+^/K^+^ ATPase causing a consequential drop of ATP levels, which is counteracted by enhancing glucose uptake and glycolysis (Magistretti and Chatton, 2005). The lactate formed by glycolysis is released by astrocytes to sustain and aid neurons (Pellerin et al., 2007). This model recapitulated in Figure 1A was introduced in the nineties and is known as the astrocyte-neuron lactate shuttle hypothesis (Pellerin and Magistretti, 1994). Of note, this idea of astrocytes “feeding hungry neurons” has been challenged, by showing that astrocytes themselves have high energy demands, concept which is generally overlooked (Dienel and Rothman, 2020).

The high cerebral metabolic rate exposes the brain to oxidative stress. About 20% of the oxygen in the body is utilized by the brain even though represents only the 2% of the body weight (Jiang and Lu, 2022). Therefore, it is critical for the brain to have a defensive mechanism against the toxic byproducts of oxidative phosphorylation which are mainly reactive oxygen species (ROS). Also in this case, the metabolic astrocytes-neuron crosstalk is important (Figure 1B) (Dringen et al., 2000). While neuronal activity inevitably induces ROS production, the antioxidant capacity of neurons is relatively weak. Consequently, astrocytes have evolved a robust antioxidant system, partially shared with neurons (Bolanos, 2016). The antioxidant defense of neurons is repressed because of continuous protein destabilization of the master antioxidant transcriptional activator, nuclear factor-erythroid 2-related factor-2 (Nrf2) (Bell et al., 2011). In contrast, Nrf2 is highly stable in neighboring astrocytes, which explains their robust antioxidant defense and resistance against oxidative stress, including biosynthesis and release of glutathione (GSH).(Habas et al., 2013; Jimenez-Blasco et al., 2015). GSH is a tripeptide composed of glutamate, cysteine, and glycine, and it serves as a crucial antioxidant and redox regulator in cells, including in the brain. Astrocytes provide neurons with the three constituent amino acids of GSH to support its local synthesis (Dringen et al., 2000).

In the last decade the phenomenon of intercellular mitochondria transfer between astrocytes and neurons has been proposed indicating a new way through which astrocytes might support neurons. This phenomenon has been reported to be occurring from astrocytes to neurons under hypoxia condition and seems to require CD38-cADPR-Ca^2+^ pathway (Figure 1C) (Hayakawa et al., 2016). However, the transmembrane protein CD38 (cluster of differentiation 38) is an inefficient cyclase. CD38 utilizes NAD^+^ as a substrate to produce by cyclase activity the second messenger cyclic adenosine diphosphate ribose (cADPR) or by hydrolase ADPR. Notably, CD38 must degrade nearly 100 molecules of NAD^+^ before forming one molecule of cADPR (Hogan et al., 2019). This suggest that the trigger events and the mechanism involved in mitochondria release might not be utilizing solely the CD38-cADPR-Ca^2+^ pathway. Nonetheless, transplanting mitochondria of astrocytic to neurons appears to improve energy production and cell viability (Hayakawa et al., 2016). Convincing evidence also indicates a movement of mitochondria from neurons to astrocytes. Davis et al. (2014) demonstrated that in the optic nerve head, large numbers of mitochondria are shed from neurons to be degraded by the lysosomes of neighboring glial cells. While the molecular mechanism of mitochondria transfer between cells requires further investigation, these initial observations point to an astrocytes-neuron mitochondria shuttling, where functional and supportive mitochondria are transferred from astrocytes to neurons and mitochondria targeted for recycling are moved from neurons to mitochondria.

Several lines of evidence indicate that, this metabolic crosstalk between astrocytes and neurons becomes dysfunctional in Alzheimer’s disease (AD). In particular, during AD, astrocytes convert into a disease associated phenotype which fail to properly support neuronal function (Liddelow et al., 2017) or even jeopardize neuronal metabolism (Crivelli et al., 2023).

While this review focuses on how astrocytes metabolically support neurons, it is important to highlight that oligodendrocytes are also essential for the function and long-term integrity of neuronal cells (Saab et al., 2013). Oligodendrocytes transfer energy metabolites such as pyruvate and lactate to neurons via specialized channels and transporters, facilitating ATP synthesis in neurons (Philips and Rothstein, 2017). They utilize “myelinic” channels and monocarboxylate transporters (MCTs) to ensure rapid delivery of these metabolites (Saab et al., 2013; Lee et al., 2012). Release of glutamate by spiking neurons stimulates N-methyl-D-aspartate (NMDA) receptors in oligodendrocytes mobilizing glucose transporter GLUT1 into the myelin compartment (Saab et al., 2016). Additionally, oligodendrocytes also enhance axonal energy metabolism through the transcellular delivery of proteins like SIRT2, which deacetylates mitochondrial proteins, thereby boosting ATP production in neurons (Chamberlain et al., 2021). These signaling mechanism underscores the importance of oligodendrocytes beyond mere structural support, highlighting their role in metabolic regulation of neuronal cells.

### 1.3 Energy metabolism disbalance in Alzheimer’s disease (AD)

AD cases are growing globally at an alarming pace, as the population ages (Cummings et al., 2023). Presently, there are estimated 50 million individuals worldwide with AD dementia, and these numbers are predicted to triple by 2050 without even counting the individuals in prodromal stage of AD (Gustavsson et al., 2023). Besides aging, type 2 diabetes mellitus is considered a risk factor in developing AD (Willette et al., 2015). Epidemiological studies have consistently shown that individuals with diabetes have an increased risk of developing AD compared to those without diabetes. The mechanisms underlying this association are complex and may involve insulin resistance, chronic hyperglycemia, impaired glucose metabolism in the brain, vascular dysfunction, and inflammation, among others (Neumann et al., 2008; Bozluolcay et al., 2016; Cater and Holter, 2022). AD patients are often more susceptible to insulin resistance in comparison to age matched controls (Domingues et al., 2021). Glucose metabolism, as assessed by positron emission tomography, is critically diminished in the hippocampus and brain cortex of familial AD subjects (Chen and Zhong, 2013). Furthermore, altered glucose uptake is observed in the brain of patients with mild cognitive impairment, which is considered to be a prodromal stage of AD (Mosconi et al., 2013). One way through which glucose becomes less bioavailable to the brain is by downregulation of GLUTs protein, which as aforementioned transports glucose from the bloodstream to the brain parenchyma (Kyrtata et al., 2021). Additionally, the use of local brain energy resources is compromised, at least partly, by oxidative damage. Oxidative stress is responsible for the downregulation of the enzymes involved in glycolysis, the tricarboxylic acid cycle and ATP biosynthesis (Butterfield and Halliwell, 2019). For a detail review on the impact of oxidative stress to glucose metabolism in AD see the exhaustive review from Butterfield and Halliwell (2019). Oxidative damage to DNA can interfere with gene transcription and proper promoter function, hindering the transcription of essential genes and generating mutations. Mitochondrial DNA, which codes for key enzymes of the ETC and oxidative phosphorylation, can also be affected, contributing to energy production impairment and mitochondrial dysfunction. For details on the mitochondria hypothesis in AD see the review of Swerdlow (2018). In brief, the mitochondrial cascade hypothesis in AD suggests that mitochondrial dysfunction is a primary event in the disease’s pathogenesis. According to this hypothesis, inherited or acquired mitochondrial defects lead to increased production of ROS, impaired energy metabolism, and the activation of apoptosis. These mitochondrial disturbances contribute to the accumulation of amyloid-*β* and tau pathology, ultimately resulting in neurodegeneration and the clinical symptoms of AD. In alignment with this hypothesis, a recent research group in the Netherlands found that the phenomenon of super-agers—individuals displaying neuropathological changes yet no cognitive decline—might be due to a high expression of a set of genes associated with the mitochondria of astrocytes (de Vries et al., 2024). However, damage to mitochondria can also result from amyloid-*β* and tau accumulation (Oliver and Reddy, 2019; Pradeepkiran and Reddy, 2020). Mitochondria maintain a dynamic balance between fusion and fission, which is critical for preserving their structure and function. In AD, mitochondrial fission is increased, while fusion is reduced, a process driven by amyloid-*β* interacting with the fission protein Drp1 (Morton et al., 2021; Manczak et al., 2011). Additionally, abnormal tau phosphorylation disrupts mitophagy by modulating key receptors such as FKBP8, which are essential for mitochondrial quality control (Isei et al., 2024).

The mitochondria cascade hypothesis raised the possibility of new therapeutic approaches focused on preventing AD-related decline of mitochondrial function, for example through exercise, diet or pharmacological manipulations to enhance brain cells bioenergetics (Atlante et al., 2022). Along these lines, UCPs play a vital role in regulating mitochondrial membrane potential, preventing excessive ROS production, alteration in neuronal activity, and in regulating calcium (Ca^2+^) homeostasis which ultimately results in the prevention of neuronal loss. In the following sections, we will discuss the neuroprotective role of UCP4 and its connection to AD.

## 2 Biological role and properties of UCP4

### 2.1 Mapping functional and binding sites within UCP4

UCPs induce a transient proton leak across the inner mitochondrial membrane, dissipating the protonmotive force and shunting oxidative phosphorylation from ATP synthesis. The dissipation of the protonmotive force is generally converted to heat. A total of five mammalian UCPs have been discovered to date, which are encoded by independent genes (UCP1 or SLC25A7, UCP2 or SLC25A8, UCP3 or SLC25A9, UCP4 and UCP5 or SLC25A14). The most studied UCP is the UCP1 (SCL25A7) that is mostly translated in brown adipose tissue. UCP1 is classically associated with non-shivering thermogenesis. In newborns and infants, who have a higher surface area-to-volume ratio and a greater propensity for heat loss compared to adults, UCP1-mediated thermogenesis in brown adipose tissue is particularly important for maintaining body temperature and thermoregulation (Johnson et al., 2023; Bienboire-Frosini et al., 2023). UCPs presumingly lack basal activity and they are activated by fatty acids (FA) and inhibited by purine nucleotides, like ATP. Cofactors like coenzyme Q (Echtay et al., 2000b) and pH shifts are also involved in regulating UCPs activity (Nicholls, 1976; Klingenberg, 1988). While the lipid binding site in UCPs has not been conclusively identified (Jing et al., 2018), it has been observed that the nucleotide binding to UCP1 requires three Arginine (R) residues in position 84, 183 and 277, which are conserved in all five members of the UCPs. These 3 R residues are located in the even number transmembrane helices (TM-2, −4 and −6). This interaction was confirmed recently by a cryo-electron microscopy study where the central cavity of UCP1 was found open to the cytosolic side and ATP was shown to bind inside this cavity, contacting TM2 and TM6 (Kang and Chen, 2023). Despite similarity in their general structures, far-UV spectra data of reconstituted recombinant UCP4 in the presence or absence of the nucleotides GDP or GTP exhibited diverse patterns, compared to UCP2 and UCP5, implying that binding of nucleotides to UCP4 might occur differently than in other UCPs (Ivanova et al., 2010). Additionally, while UCP2 transported protons at the highest rate in the presence of arachidonic acid, UCP4 and UCP5 achieved their maximum proton transport rates in the presence of oleic acid (Hoang et al., 2015). This test was run among saturated FA (lauric acid and palmitic acid) unsaturated FA (Oleic acid, linolenic acid and linoleic acid) and bulky unsaturated FA (arachidonic acid and docohexaneoic acid). The proton transport of UCPs in presence of sphingoid base lipids has not been studied yet, despite recent studies connecting sphingolipid metabolism to mitochondria homeostasis (Sheridan and Ogretmen, 2021; Bionda et al., 2004).

**Figure 2:**
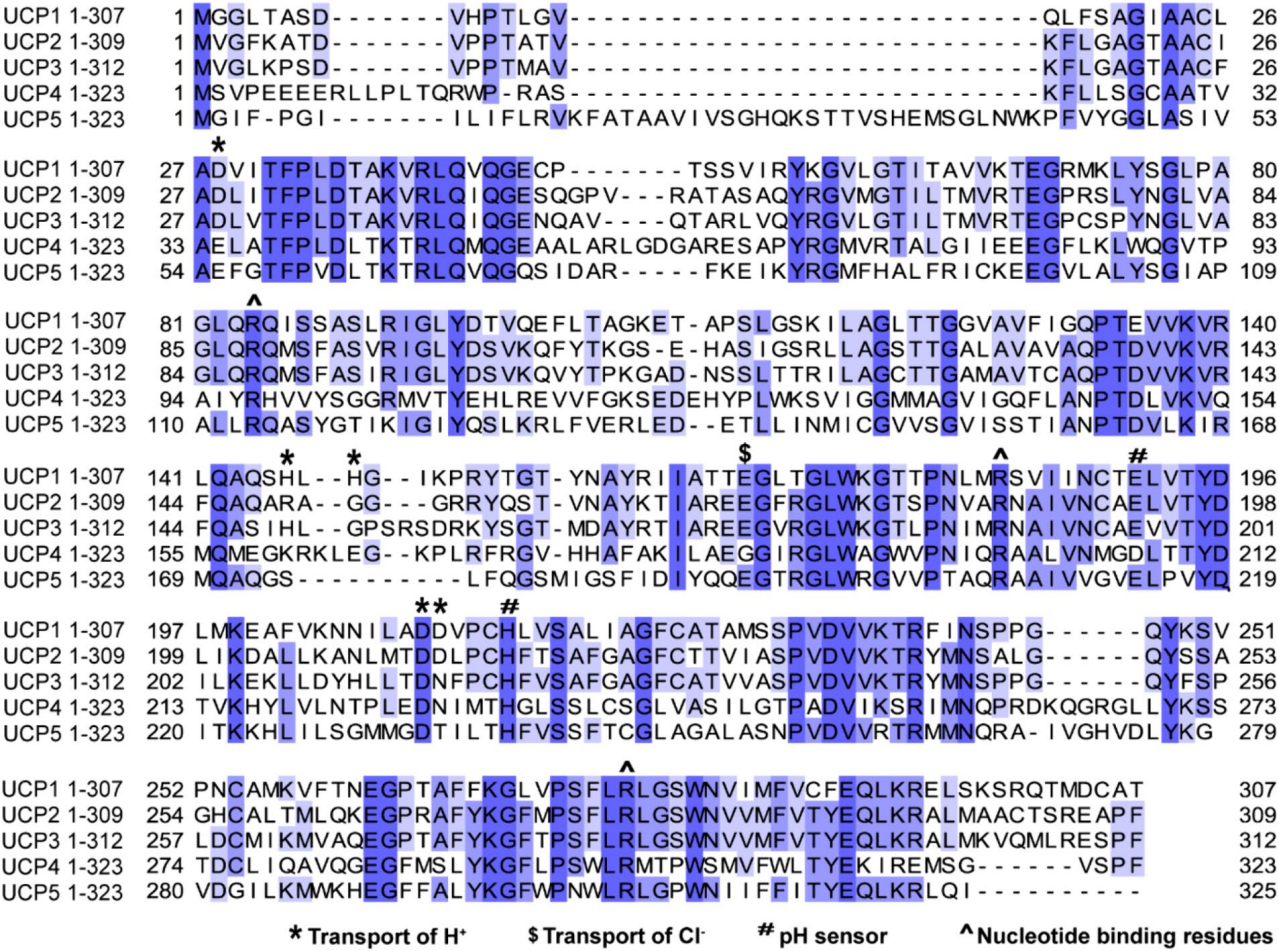
Sequence alignment of UCPs proteins highlighting percentage of identity and mapping the functional residues. The human protein sequences of UCP1 (Uniprot P25874), UCP2 (Uniprot Uniprot P55851), UCP3 (Uniprot P55916), UCP4 Uniprot (O95847) and UCP5 (Uniprot O95258) were aligned using Jalview software 2.11.3.2 with T-Coffee server, visualizing similarity among sequences with percentage of identity option which colors the residues according to the (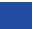 > 80%, 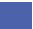 >60%, 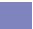 > 40%, 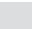 < 40%). Residues which have been implicated by site-directed mutagenesis in transport of H^+^, sensing pH shifts, transport of Cl^−^ or interacting with nucleotide are highlighted across sequences.

In Figure 2 recapitulates functional mapping of critical residues in aligned UCPs that are discussed below in more detail. Site-directed mutagenesis on UCP1 highlighted that the carboxyl group of the Aspartic Acid (D) in position 27 (D27) plays a critical role in proton transport (Echtay et al., 2000a). This carboxyl group is within the transmembrane region of the first helix, and it is mostly conserved between UCP members. In UCP4, D27 is substituted with the Glutamic acid (E) which is also a negatively charged residue. Substitution of D27 with E27 maintained UCP1 capacity to transfer protons, even though less efficiently (Echtay et al., 2000a). This is in line with a later study where UCP4 showed a lower H^+^ transport rate compared to UCP2 and UCP5 (Hoang et al., 2012). Moreover, site-directed substitution of the carboxyl group in position D210 with an uncharged amino acid, decreased the H^+^ transport of about 80%, uncovering a second critical residue associated to UCP proton transport (Echtay et al., 2000a). D210 is located in proximity of the Histidine (H) in position 214 (H214), which, together with E190, are pH sensors regulating the binding of nucleotides to UCPs. The E190 is the gatekeeper which controls the access of the nucleotides to the binding pocket by protonation (Klingenberg, 2017; Echtay et al., 1997). Meanwhile, the protonation of 214 determines the size of the pocket, where nucleotides bind, facilitating interaction with di- or triphosphate nucleotide (Klingenberg, 2017; Echtay et al., 1998). Also, a pair of H residues in position 145 and 147, which are missing in UCP4, were identified to be fundamental for UCP1 H^+^ transport (Bienengraeber et al., 1998).

UCPs have been shown to conduct anions like Cl^−^, Br^−^ and NO_3_^−^ as well (Huang and Klingenberg, 1996). In UCP1 substitution of E167, which is located on the matrix side of the membrane, with Glutamine (Q) reduced drastically Cl^−^ transport without affecting H^+^ transport (Echtay et al., 2000a). This suggests that proton and anion transport are independent in UCPs. The exact mechanism through which UCPs conduct H^+^ and Cl^−^ is still unclear. To date, several hypotheses have been proposed: i) biphasic two-state model for proton transport (Ardalan et al., 2021), ii) FA protonophore model (Garlid et al., 1996) and iii) the proton buffering model (Garlid et al., 1998). The biphasic two-state model proposes that UCPs mediate proton transport through two distinct phases. In the first phase, UCPs undergo a cycle of protonation and deprotonation of specific amino acid residues within the protein. In UCP2, where this model was initially tested, protons were proposed to interact with three specific arginine residues (R88, R185, and R279) and an aspartate (D28) that are present close to the center of the protein and can induce a set of conformational changes which facilitate the proton to be released in the matrix side. In the second phase, the protonation/deprotonation cycle induces conformational changes in the UCP protein, leading to the interconversion between cytoplasmic and matrix states (Ardalan et al., 2021).

The FA protonophore model suggests that UCPs mediate proton transport indirectly through the cycling of FA across the inner mitochondrial membrane, thus behaving as cycling protonophores (Garlid et al., 1996).

According to proton buffering model, UCPs facilitate proton transport across the inner mitochondrial membrane by functioning as channel based on the discovery of two H^+^-conducting histidine residues in UCP1, (H145, and H147) (Bienengraeber et al., 1998). The proton buffering model may coexist with the FA protonophore model and the biphasic proton transport mechanism.

**Figure 3:**
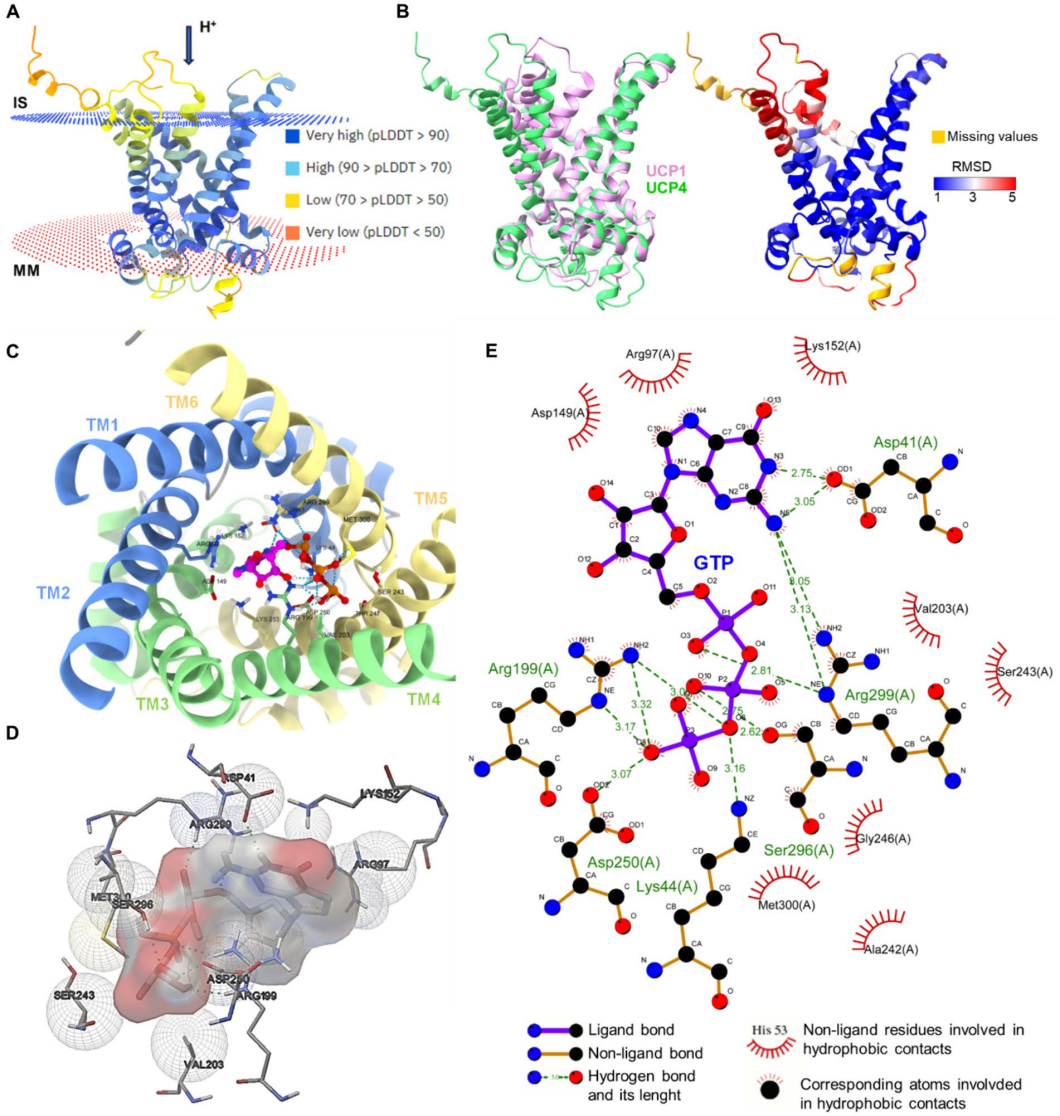
*In silico* modeling of UCP4 binding site for purine nucleotide triphosphates. **(A)** AlphaFold protein structure prediction for UPC4 (AF-O95847-F1-model, Uniprot ID O95847) colored by confidence score (pLDDT) and showing membrane position calculated using PPM 3.0 web server. IS, intermembrane space; MM, mitochondria matrix. **(B)** The superimposition of UCP1 (RCSB_ID 8HBW) with UPC4 (AF-O95847-F1-model) using UCSF ChimeraX version: 1.7 matchmaker which was then rendered by Root Mean Square Deviation (RMSD) attribute (left), the residues of UCP4 with high correspondences with UCP1 are represented in blue (RMSD <1Amstrong) (right). **(C)** Cytoplasmic view of the GTP-UCP4 interaction with the helix colored. **(D)** 3D side view of GTP interaction with binding residues of UCP4 (AF-O95847-F1-model) predicted with AutoDock4 version 4.2.6. **(E)** 2D interaction diagram of GTP-UCP4 interaction using LigPlot^+^ version 2.2 results representing 2D.

In Figure 3, we used *in silico* prediction modeling to determine the potential binding site of purine nucleotide [i.e., Guanosine-5′-triphosphate (GTP)] to UCP4, starting from the AlphaFold structure prediction of UCP4. The binding site interaction analysis confirmed the importance of the conserved residues R97, R199, and R299 for GTP binding to UCP4.

### 2.2 Tissue distribution and function of UCP4

**Figure 4:**
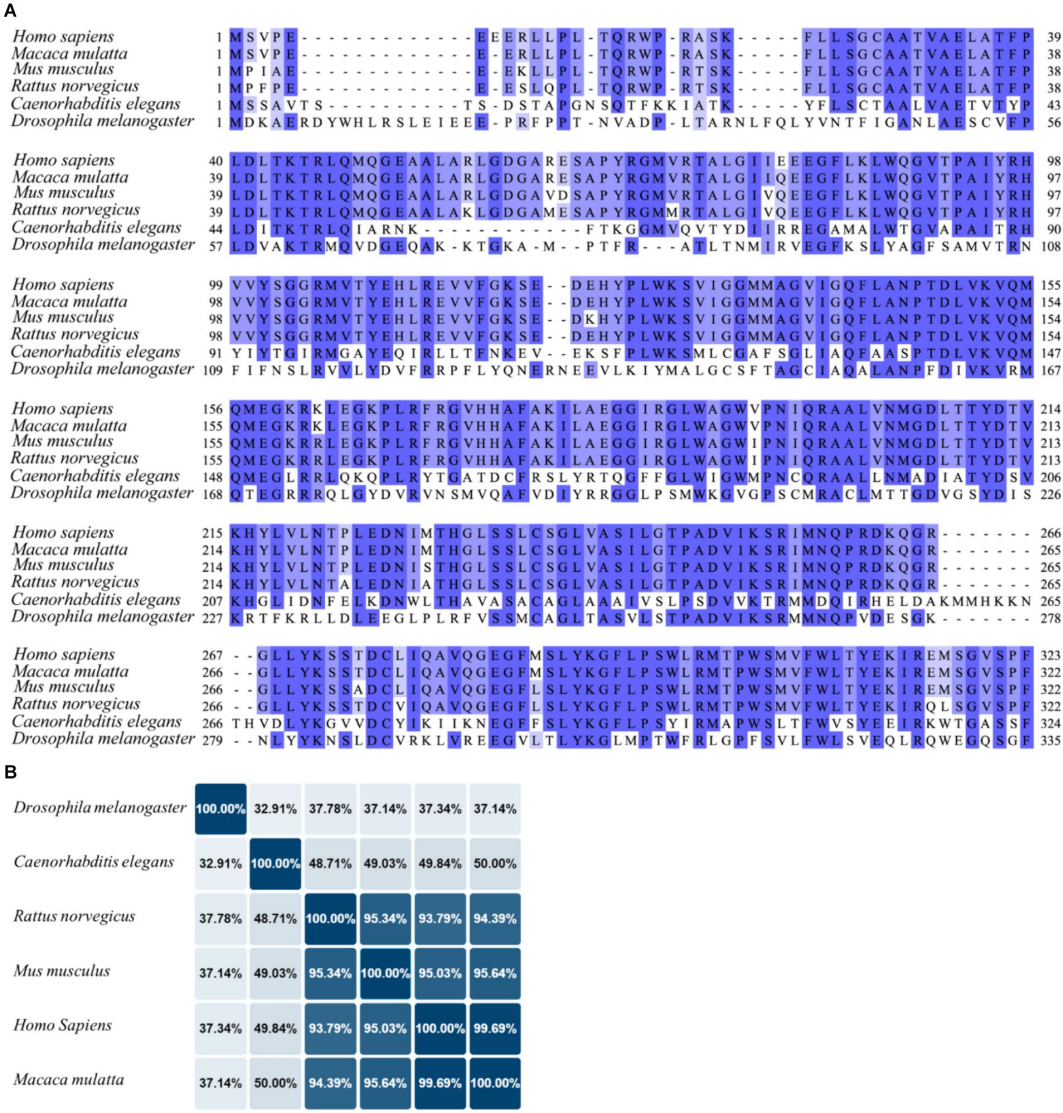
Sequence alignment of UCP4 in different species. **(A)** The UCP4 protein sequences from humans (*Homo sapiens*, UniProt ID: O95847), monkeys (*Macaca mulatta*, UniProt ID: F6QG76), mice (*Mus musculus*, UniProt ID: Q9D6D0), rats (*Rattus norvegicus*, UniProt ID: A6JJ30), nematodes (*Caenorhabditis elegans*), and flies (*Drosophila melanogaster*, UniProt ID: Q9VMK1), were aligned using Jalview software 2.11.3.2 with T-Coffee server, visualizing similarity among sequences with percentage of identity option which colors the residues according to the (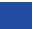 > 80%, 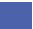 >60%, 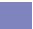 > 40%, 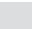 < 40%). **(B)** The identity matrix between UCP4 sequences was generated using UniProt.

Even though one of the less studied UCPs, UPC4 resulted to be the ancestral UCP from which other mammalian and plant UCPs probably diverged (Hanak and Jezek, 2001). In mammals, the protein sequences of UCP4 analogues are highly conserved, suggesting that the biological functions of this protein are largely preserved across mammalian species (Figure 4A). For instance, an identity matrix generated using UniProt alignment shows that the homology between human and mouse UCP4 exceeds 95%, indicating that these sequences are nearly identical (Figure 4B). In contrast, the homology of UCP4 analogues in *C. elegans* and *Drosophila melanogaster* with the human UCP4 is lower, at 49 and 37%, respectively, but still significant (Pearson, 2013). UCP4 was firstly described in 1999 as brain specific mitochondrial protein with uncoupling function (Mao et al., 1999). For this reason, UCP4 has been defined as neuronal UCP, together with UCP2 and UCP5. Unlike UCP1, these proteins (UCP2, UCP4, and UCP5) are not constitutive uncouplers and are not crucial for non-shivering thermogenesis. Of notice, while UCP2 and UCP5 have a wider tissue distribution, recent studies using immunolabelling approaches confirmed that UCP4 is almost exclusively expressed in the brain, with the highest protein levels found in the cortex (Smorodchenko et al., 2009). Cellular expression of UCP4 was localized in neurons and astrocytes and it was absent in microglia (Smorodchenko et al., 2009). Interestingly, UCP4 is highly expressed during early embryogenesis in mice, day 12–14, a critical period for neuronal development and differentiation. Conversely, the UCP4 protein levels decline with aging (Mao et al., 1999; Smorodchenko et al., 2009).

*In vitro* and *in vivo* data suggest that UCP4 might have broader functions than solely depolarizing mitochondria electrochemical potential. Chu et al. (2009) reported that overexpression of UCP4 in human neuroblastoma cells conferred protection against mitotoxic signals such as MPP+ (1-methyl-4-phenylpyridinium), a compound that interferes with oxidative phosphorylation by inhibiting mitochondrial complex I. This neuroprotection mediated by UCP4 against induced oxidative stress did not involve canonical uncoupling effects, but rather it was created by a fine tuning of H^+^ flow in both direction of the mitochondria inner membrane, which preserved ATP synthesis (Chu et al., 2009). This confirms the work of Klotzsch et al. (2015), which concluded that UCP4 cannot uncouple phosphorylation from proton pumping because of its position in the inner mitochondrial membrane distant from the F_0_F_1_-ATP synthase. Klotzsch et al. (2015) further observed that UCP4 is preferentially localized in close vicinity to the mitochondrial voltage-dependent anion channel (VDAC). Moreover, UCP4 exhibits the lowest average proton transport among the neuronal UCPs, when reconstituted in liposomes (Hoang et al., 2015). Nonetheless, UCP4 can effectively shortcut excessive transmembrane proton gradients for regulating ROS production (Klotzsch et al., 2015). Characterization of the promoter region of human UCP4 identified a potent nuclear factor (NF)-κB response element binding site within this region (Ho et al., 2010). Therefore, UCP4 expression is significantly regulated via activation and inhibition of NF-κB signaling and seems to act as one of the effector of the NF-κB c-Rel pro-survival pathway against oxidative stress (Ho J. W. et al., 2012). So, it is not surprising that UCP4 confers neuroprotection under several cell stress conditions. For instance, UCP4 protected neurons from oxidative stress by regulating mitochondrial Ca^2+^ homeostasis and sensitivity, during induced depletion of Ca^2+^ in the endoplasmic reticulum (ER) (Chan et al., 2006). Mitochondria buffers Ca^2+^ during Ca^2+^ release from the ER or Ca^2+^influx from the plasma membrane, preventing cytosolic Ca^2+^ overload. At the same time, excessive Ca^2+^ levels in the matrix of mitochondria results in an increase of ROS that promotes apoptosis (Chen et al., 2017). UCP4 stably reduced neuronal mitochondria membrane potential, avoiding mitochondria overload during Ca^2+^ store depletion-induced toxicity (Chan et al., 2006). Conversely, the depletion of UCP4 expression in primary hippocampal neurons culminates in mitochondrial Ca^2+^ increase and subsequent cell death (Liu et al., 2006). This raises the question on how UCP4 senses or interferes with Ca^2+^ signaling. This topic is further discussed in the next subsection.

Another mechanism through which UCP4 is preserving neuronal function is by increasing ATP synthesis by interacting with complex II (Ho P. W. et al., 2012). Neuron-like-cells stably expressing UCP4 exhibited higher oxygen consumption and increased ATP production compared to controls. Additionally, in these cells, complex II activity was significantly higher but not the activity of complex I and IV. This highlights a unique role of UCP4 as a potential regulatory target to modulate mitochondrial Complex II and ATP output to preserve neurons against energy crisis (Ho P. W. et al., 2012).

Interestingly, overexpression of UCP4 in astrocytes seems also to achieve neuroprotection (Perreten Lambert et al., 2014). This study shows that overexpressing UCP4 in astrocytes reduces mitochondrial electrical potential together with oxygen peroxide release, and most importantly it enhanced secretion of lactate which likely mediated pro survival effect in neurons. Astrocytes produced more lactate because of enhanced glycolysis, which was triggered by the diminished levels of mitochondrial respiration. However, UCP4 did not increase lactate production in response to glutamate (Perreten Lambert et al., 2014). This is compatible with an additional hypothesis, where lactate release is increased in UCP4 overexpressing astrocytes due to changes in plasma membrane permeability to lactate. It has been, shown that during thermogenesis in brown adipocytes the plasma membrane increases its permeability to lactate by upregulating monocarboxylate transporters MCT1 and MCT4, which are lactate transporters (Petersen et al., 2017).

### 2.3 Neuronal Ca^2+^ sensor 1 (NCS1) and mitochondrial UCP4

**Figure 5:**
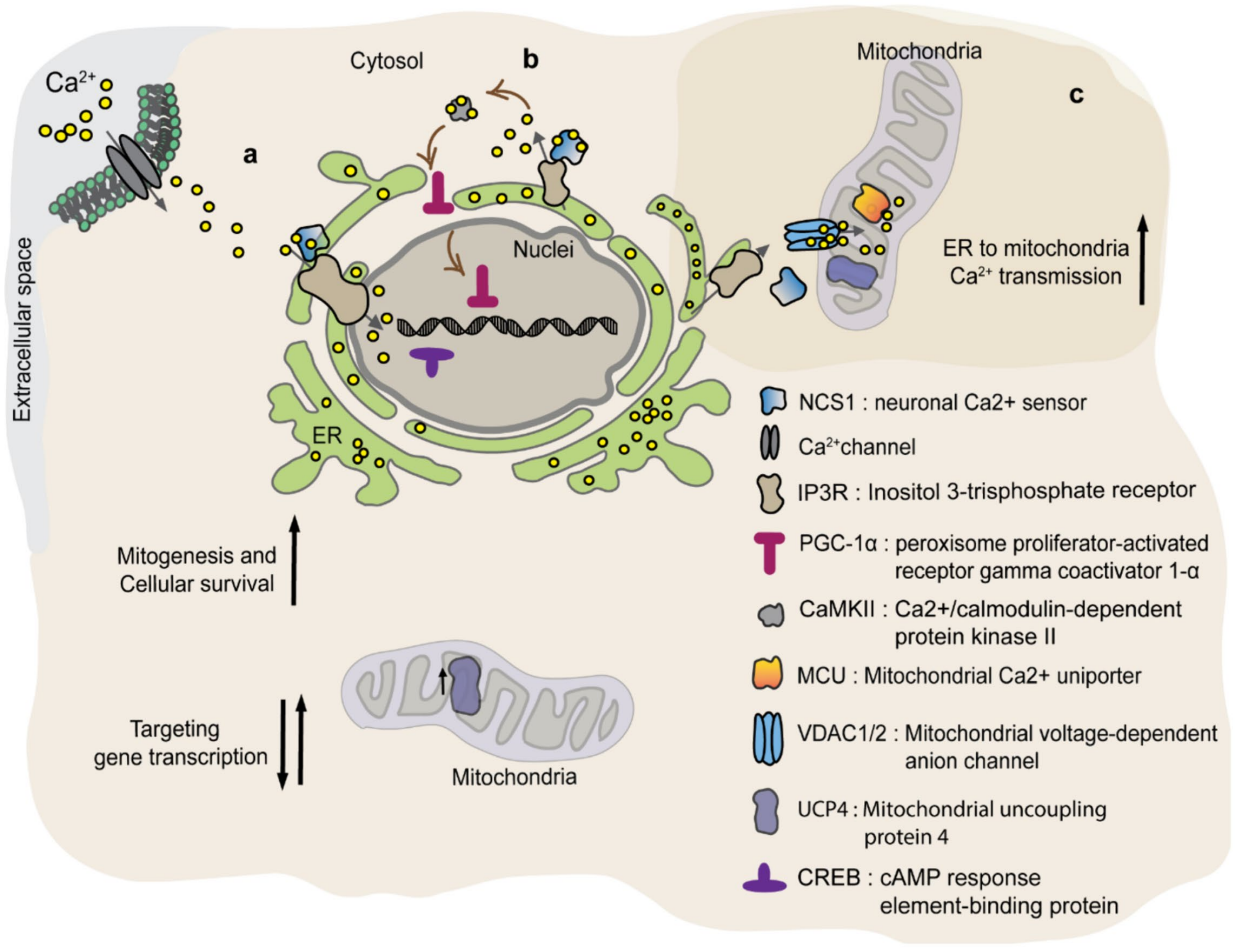
NCS1 regulates UCP4 protein levels and mitochondrial Ca^2+^ homeostasis. NCS1 might regulate UCP4 gene expression in two ways: a) via a cytosolic Ca^2+^ concentration dependent manner or b) independent from cytosolic Ca^2+^. (a) Cytosolic Ca^2+^ activates NSC1 initiating interaction with IP3R located in the perinuclear region. IP3R increases nuclear Ca^2+^ targeting transcription factors like CREB. (b) NSC1 interacts with IP3R inducing Ca^2+^ release from ER (endoplasmic reticulum) which activates CaMKII. CaMKII promotes PGC-1α translocation to the nuclei, where it regulates mitochondrial biogenesis and cellular survival mechanisms. (c) NCS1/UCP4 can regulate mitochondrial Ca^2+^ stabilizing its uptake. Efficient Ca^2+^ transfer from ER to mitochondria at contact sites requires orchestrated activation of proteins involved in ER Ca^2+^ release (NCS1/IP3R) and mitochondrial Ca^2+^ uptake (VDAC1/2 and MCU). While activation of NCS1/IP3R/VDAC1 and MCU provides Ca^2+^ transfer from ER to mitochondria, UCP4 regulates the ability of mitochondria to buffer Ca^2+^ via modulation of mitochondrial membrane potential and its pH.

A biological link was found between the Ca^2+^ sensor, neuronal Ca^2+^ sensor 1 (NCS1), and UCP4 (Simons et al., 2019). NCS1/frequenin belongs to the family of neuronal calcium sensors. Neurons express a wide variety of calcium sensors characterized by different Ca^2+^ binding affinities. NCS1 contains the EF hand Ca^2+^ binding motif which is activated by sub-micromolar changes of Ca^2+^ concentration. This allows rapid cellular responses to small fluctuations in cytosolic Ca^2+^ levels (Burgoyne, 2007). Genetic knock-out (KO) of NCS1 in mice resulted in a lower expression of mitochondrial UCP4 in dopaminergic neurons of the substantia nigra, which are highly vulnerable to Parkinson’s disease (Simons et al., 2019). This suggests that UCP4 gene transcription or its mRNA stabilization might be regulated by NCS1 (Simons et al., 2019). A similar correlation was observed between NCS1 and other UCPs, like UCP2 and UCP3 in cardiomyocytes. In brief, NCS1 KO myocytes displayed reduced expression of mitochondrial proteins involved in oxidative phosphorylation as well as UCPs, causing impaired mitochondrial respiration and increased susceptibility to oxidative stress (Nakamura et al., 2016). How NCS1 affects the expression and function of mitochondrial proteins is not completely understood. The perinuclear localization of NCS1 and its ability to regulate nuclear Ca^2+^ signaling, suggest a rapid modulation of mitochondrial UCP4 levels by NCS1 (Figure 5a) (Simons et al., 2019; Nakao et al., 2015; Xu et al., 2018; Guzman et al., 2010). As a response to the rise in cytosolic Ca^2+^, NCS1 binds to inositol trisphosphate receptor (IP3R) receptors located in the perinuclear region and affect Ca^2+^ dependent gene expression via modulation of transcription factors as cAMP response element-binding protein (CREB) (Nakao et al., 2015). Alternatively, NSC1 interacts with IP3R activates signaling pathways such as PGC-1α and/or PI3-K-Akt, which regulate mitochondrial biogenesis and cellular survival mechanisms (Figure 5b) (Nakamura et al., 2016; Nakamura et al., 2019). Besides NSC1 controlling UCP4 expression, recent publication suggests a functional association between the two proteins. Efficient Ca^2+^ transfer from ER to mitochondria at contact sites requires orchestrated activation of proteins involved in ER Ca^2+^ release (NCS1/IP3R) and mitochondrial Ca^2+^ uptake [VDAC1/2 and mitochondrial Ca^2+^ uniporter (MCU)] (Angebault et al., 2018). The mechanism is recapitulated in Figure 5c. While activation of NCS1/IP3R/VDAC1 and MCU provides Ca^2+^ transfer from ER to mitochondria, UCP4 regulates the ability of mitochondria to buffer Ca^2+^ via modulation of mitochondrial membrane potential and its pH. Thus, NCS1-UCP4 interaction seems to be involved in proper Ca^2+^ transmission from ER to mitochondria, preventing mitochondrial Ca^2+^ overload and cellular oxidative stress.

## 3 The impact of UCP4 in the pathophysiology and treatment of AD

### 3.1 Evidence of UCP4 involvement in AD pathology

As mentioned above, oxidative stress and mitochondrial dysfunction are becoming recognized not only as critical molecular features of AD but also to occur at early stages of the disease. Gene expression analysis, performed on the prefrontal lobe of AD patient, manifesting different level of severity determined by Braak Stage, revealed reduce levels of respiration chain proteins, while markers for oxidative stress such as nitric oxide synthase were increased (de la Monte and Wands, 2006). Interestingly, the levels of the neuronal UCPs, UCP2, 4 and 5 were significantly reduced in postmortem AD brain tissue (de la Monte and Wands, 2006). In 2016, it was reported that a specific UCP4 variant contributes to the risk of developing AD (Montesanto et al., 2016). The authors observed that the UCP4 variant rs9472817 increased 1.50-fold the risk to develop AD, when affecting one allele, and of 2.24-fold when variation was present in both alleles. This gene dosage dependent effect was true for both late onset and familial AD. Another intriguing aspect of this study highlighted is that UCP4 variant rs9472817 seems to be biologically associated to apolipoprotein E-ε4 (APOE4) alleles, since the highest risk was found for patients carrying APOE4 and the UCP4 variant. What remains unanswered is which type of functional change does rs9472817 UCP4 variant cause to the protein and which biological link exist between UCP4 and APOE4. Answering these questions is now even more relevant considering the new findings where APOE4 homozygosity might be consider as a distinct genetic form of AD (Fortea et al., 2024).

In AD, UCP4 is downregulated probably in response to pro-inflammatory signals, like protein glia maturation factor or nitric oxide, released by glia cells (Thangavel et al., 2017). The authors proposed that downregulation of UCP4 is probably downstream to NF-κB p65 signaling. A*β* oligomers, which are produce in the early phase of the AD, are known to activate NF-κB in neurons and glia via the Toll-like receptor 4 (Kaltschmidt et al., 1997). Additionally having one or two APOE4 alleles, which is a strong genetic risk factor for the development AD (Corder et al., 1993), primes microglial cells toward a phagocytic and proinflammatory state in normal aging as well as in AD (Serrano-Pozo et al., 2021).

### 3.2 Targeting UCPs/mitochondria metabolism during AD

Cho et al. (2016) found that *Caenorhabditis elegans* (*C. elegans*) defective of UCP4 exhibited increased mitochondrial membrane potential, which was associated with enhanced neuronal defects during aging. Conversely, overexpression of UCP4 mitigated neuronal defects. Additionally, the UCP4-knock-out showed resistance to the pathogen *Staphylococcus aureus*, possibly due to high levels of ROS. However, susceptibility to the pathogen increased with age in the UCP4- knock-out, suggesting a complex interplay between ROS and immunity. These findings highlight the role of UCP4 in regulating mitochondrial function, neuronal health, and immune response in *C. elegans*, during aging.

Recently, our group discovered that overexpression of UCP4 in astrocytic mitochondria prevented multilevel dysfunction in a mouse model of AD (Rosenberg et al., 2023). Viral particles delivering the murine variant of UCP4 in astrocytes, via the GFAP promoter, were injected into 2-month-old triple-transgenic AD mice (3 × Tg-AD). The 3 × Tg-AD mice, harboring PS1M146V, APPSwe, and tauP301L transgenes, manifest slow but progressive development of amyloid-*β* plaques and tangles (Oddo et al., 2003). Synaptic dysfunction, including deficits in long-term potentiation, occurs in an age-related manner but precedes plaque and tangle pathology (Oddo et al., 2003). Metabolomic analysis of the hippocampus showed that 1–2 month post-viral injection, astrocytic overexpression of UCP4 normalized a series of metabolites related to the TCA cycle, glycolysis, and amino acid metabolism (Rosenberg et al., 2023).

Intriguingly, long-term overexpression of UCP4 in astrocytes prevented dendritic loss and aberrant electrical properties of subicular neurons, hippocampal atrophy, and decline in spatial memory in the 3xTg-AD mouse model (Rosenberg et al., 2023). Computational modeling further supported our experimental observations by predicting a reduction in neuronal Ca^2+^ entry at low voltages in mice treated with UCP4. This reduction was attributed to the modulation of T-type voltage-gated calcium (Cav) channels, which play a crucial role in burst activity generation in subicular neurons (Aguado et al., 2016). T-type Cav channels are activated at low membrane potentials (around −70 mV) and participate in the generation of burst activity in subicular neurons (Joksimovic et al., 2017). The reduction in Ca^2+^ influx at low voltages in 3xTg-AD mice suggested by computational modeling strongly correlates with experimental data. Firstly, the expression levels of T-type Cav3.1 channels decline during aging and AD in both humans and mouse models (Rice et al., 2014). Furthermore, pharmacological downregulation of T-type Cav channels increased A*β* production, while stimulating their activity improved cognitive function in 3xTg-AD mice, indicating a biological link between T-type Cav function and AD-associated memory dysfunction (Moriguchi et al., 2012; Green et al., 2011). More broadly, dorsal subiculum-specific knockdown of the T-type Cav3.1 channel led to spatial memory deficits in mice, confirming the relevance of T-type Cav channels in memory (Joksimovic et al., 2023).

Additionally, Ca^2+^ influx via voltage-gated T-type Ca^2+^ channels was shown to modulate Ca^2+^-sensitive K^+^ channel activation, leading to membrane potential hyperpolarization and a consequential decrease in neuronal activity (Poetschke et al., 2015). Thus, the interplay of voltage-gated Ca^2+^ and Ca^2+^-sensitive K^+^ channels serves as a negative feedback mechanism, leading to the reduction of neuronal activity and activity-related Ca^2+^ entry. Besides Cav channels, Ca^2+^-sensitive K^+^ channels are also modulated by Ca^2+^ released from intracellular Ca^2+^ stores (Stutzmann et al., 2006). Along these lines, experimental data and computational modeling suggested a reduction in K^+^ conductance in UCP4-overexpressing 3xTg-AD mice. Additionally, subicular neurons from UCP4-overexpressing 3xTg-AD mice were characterized by a smaller afterhyperpolarization compared to control virus-expressing 3xTg-AD mice. This finding, together with computational modeling, suggests that UCP4 might correct neuronal activity via modulation of Ca^2+^-sensitive K^+^ channels.

The implications of our findings extend beyond AD, as emerging evidence suggests the therapeutic potential of uncoupling mitochondria in astrocytes for other brain disorders such as depression. Du et al. demonstrated that UCP2 deficiency exacerbates depressive symptoms in mice, leading to impaired neurogenesis and increased astrocyte loss (Du et al., 2016). Moreover, UCP2 knockout (KO) mice exhibited enhanced NLRP3 inflammasome activation, mitochondrial dysfunction, ROS production, and activation of the TXNIP-NLRP3 pathway in astrocytes (Du et al., 2016). Conversely, UCP2 overexpression in astrocytes partially reversed these effects, highlighting the role of UCP2 in ameliorating depression-related mechanisms.

Transitioning to the next subsection, we will explore the rising field of pharmacology aimed at specifically activating UCPs to harness their potential therapeutic benefits.

### 3.3 The rising of a new pharmacology to specifically activate UCPs

The two compounds 2,4 dinitrophenol (DNP) and cyanide-4-(trifluoromethoxy)phenylhydrazone (FCCP) have been indispensable experimental tools for controlling mitochondrial respiration by acting as protonophores. However, in 2022 it was discovered that their mechanism of action might have been at least partly misunderstood. Bertholet et al. (2022) found, by using patch-clamp techniques, that DNP and FCCP have a preference towards the mitochondrial inner membrane (IMM) where they initiate proton currents. Conversely, DNP and FCCP induced proton currents were not detected in plasma membrane preparations, where UCPs are absent. When UCP1 inhibitors like guanosine 5′-diphosphate were introduced to the set-up H^+^ currents were strongly supressed in the IMMs. In addition, the induction of H^+^ currents by DNP and FCCP was also absent in UCPs deficient IMMs. Thus, protonophoric uncouplers are synthetic activators of H^+^ currents and require UCPs for their mode of action. It was further discovered that, in the UCP1 protein the binding site of DNP is in proximity to that of ATP, suggesting that DNP competes and can displace ATP blockade, resulting in the induction of the proton-conducting activity of UCPs.

**Figure 6:**
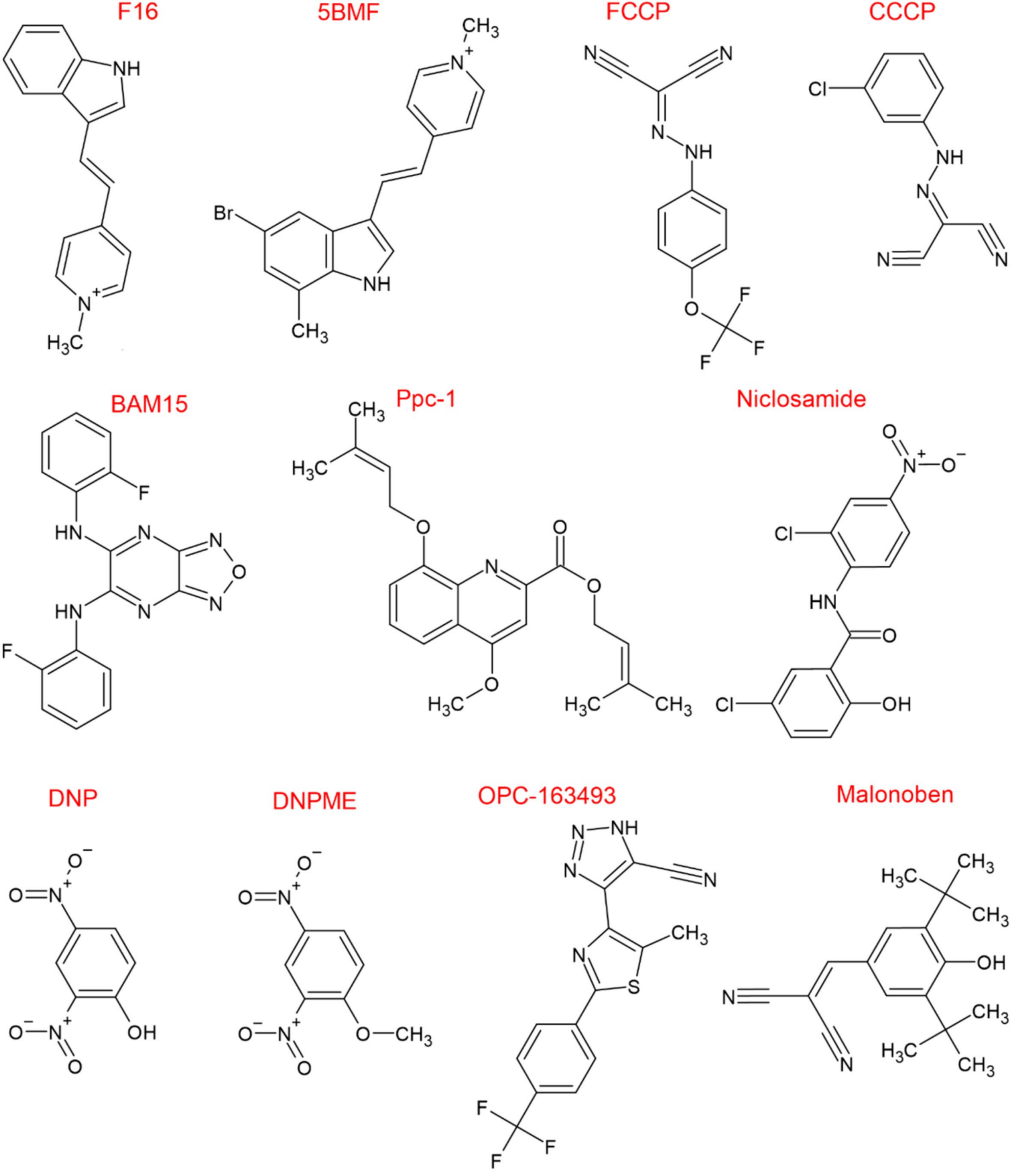
Chemical structure of known uncouplers. Chemical structures were drawn using ChemSketch (Freeware) 2023.1.2.

In Figure 6 we report the chemical structure of the known uncouplers. Protonophoric uncouplers have shown to be effective in combating obesity, diabetes, and steatosis in animal models (Perry et al., 2015). Furthermore, DNP reduces postnatal hypoxia by preventing ROS formation in the brain (Villa et al., 2023). Despite the encouraging data, the clinical potential of uncouplers for treating human disease is limited due to indiscriminately increasing H^+^ conductance across all biological membranes, causing adverse side effects (Grundlingh et al., 2011). However, safety and efficacy of DNP may be achieved by designing control release system of the drug that maintain plasma levels of DNP in the range 1 to 5 μM (Perry et al., 2015). Additionally, now that the DNP binding pocket in UCPs has been identified new DNP derivatives could be designed with modified pharmacokinetics and pharmacodynamics to minimize off-target effects (Kang and Chen, 2023). Interestingly, by screening small molecule chemical library Kenwood et al. (2014) identified and validated a novel mitochondrial protonophore uncoupler named BAM15 that does not depolarize the plasma membrane. Compared to FCCP, an uncoupler of equal potency, BAM15 treatment of cultured cells was shown to stimulate a higher maximum rate of mitochondrial respiration and to be less cytotoxic. Importantly, BAM15 is bioactive *in vivo* (Kenwood et al., 2014). Recent research suggests that BAM15 holds promise in alleviating neurodegeneration and extending lifespan in aged *Caenorhabditis elegans* (Cho et al., 2022). Treatment with BAM15 or DNP, significantly reduces neuronal defects and preserves sensory functions in aging worms. Interestingly, even mutants lacking UCP4 respond positively to BAM15 by extending their lifespan (Cho et al., 2022). Surprisingly, BAM15 like other chemical unclouplers such as niclosamide and CCCP has inhibitory effect on NLRP3 inflammasome activation through inhibiting NFκB nuclear translocation suggesting potential anti-inflammatory effects (Hu et al., 2021). The finding of the mode of action of protonophore and their binding pocket in UCPs might pave the way for the development of less toxic, more potent and specific mitochondrial uncouplers that will hopefully reignite interest in pharmacological uncoupling for the treatment of diseases that are associated with altered mitochondrial function.

## 4 Conclusion and future perspectives

The exploration of UCPs’ function in the brain is still in its early stages, yet a mounting body of evidence underscores their critical role in regulating brain metabolism and influencing brain function. In this comprehensive review, we explored the bioenergetic interconnection between astrocytes and neurons, and the emerging significance of UCPs in AD. Within astrocytes, UCPs play a pivotal role in modulating inflammatory responses via NLRP3 inflammasome activation and facilitating neurotransmission. Interestingly, the UCP4-knock-out showed resistance to the pathogen *Staphylococcus aureus*, possibly due to high levels of ROS. However, susceptibility to the pathogen increased with age in the UCP4- knock-out, suggesting a complex interplay between UCPs protein levels, ROS and immunity. The mechanism of this interplay remains vastly unexplored. The downregulation of UCPs in AD pathology highlights its potential as a therapeutic target for mitigating the progression of critical aspects of the disease. Indeed, the work of Rosenberg et al. (2023) provided compelling data that mild uncoupling via overexpression of UCP4 in astrocytes can prevent dendritic loss, aberrant neuronal activity, and cognitive decline in AD mouse models. However, this data also underscores how our knowledge about the function of UCPs in the brain remains limited, such as the differing functions of UCPs depending on brain cell type. The next significant step forward will rely on the development of cell-specific UCPs knockouts.

Furthermore, the discovery of the mode of action of DNP and FCCP and their binding pocket in UCPs offer exciting opportunities for the development of new less toxic and isoform specific UCP modulators of mitochondrial bioenergetics that may be employed in the clinic. The small molecule BAM15, a relatively new uncoupler, already demonstrated less off-target unwanted effects and yet promising efficacy in combating neurodegeneration in aged *C elegans*.

In summary, the elucidation of the role of UCPs, particularly UCP4, in neurodegenerative disorders represents a significant advancement in our understanding of brain energy metabolism and offers promising prospects for the development of novel therapeutic strategies for AD and other neurological conditions. Continued research in this field is essential to translate these findings into effective treatments that can improve the lives of patients affected by these devastating diseases.
